# Data on antibiogram and resistance genes harboured by *Salmonella* strains and their Pulsed-field gel electrophoresis clusters

**DOI:** 10.1016/j.dib.2018.01.098

**Published:** 2018-02-03

**Authors:** Li-Oon Chuah, Ahamed-Kamal Shamila Syuhada, Ismail Mohamad Suhaimi, Tajudin Farah Hanim, Gulam Rusul

**Affiliations:** a Food Technology Division, School of Industrial Technology, Universiti Sains Malaysia, 11800 Minden, Penang, Malaysia; b Food Safety and Quality Control Laboratory, Km 1, Jalan Abi Tok Hashim, 01000 Kangar, Perlis, Malaysia

**Keywords:** Multidrug resistance, *Salmonella*, Pulsed-field gel electrophoresis, Antibiotic resistance gene, Poultry

## Abstract

This article describes the Pulsed-field gel electrophoresis clustering of the predominant *Salmonella* strains (*Salmonella* ser. Albany, *Salmonella* ser. Brancaster, and *Salmonella* ser. Corvallis) isolated from poultry and processing environment in wet market and small-scale processing plant in Penang and Perlis, the northern states of Malaysia. Agar disk diffusion assay was performed to determine the phenotypic antibiotic resistance of these *Salmonella* strains. The most common antibiograms among the three predominant *Salmonella* serovars were reported. The presence of integrase genes and antibiotic resistance genes conferring to resistance against β-lactams, aminoglycosides, tetracyclines, quinolones, sulphonamides and chloramphenicol, was detected via PCR amplification.

**Specifications Table**

**Table:** 

Subject area	*Microbiology*
More specific subject area	Foodborne pathogen
Type of data	Table
How data was acquired	Antibiograms were determined using the agar disk diffusion assay. Antibiotic resistance genes were detected using PCR (TProfessional Standard Gradient96 Thermocyler, Biometra, Germany). Typing of the *Salmonella* strains was performed using Pulsed-field gel electrophoresis (PFGE) (Biorad CHEF Mapper system, Hercules, CA) coupled with Bionumerics software version 7.0 (Applied Maths, Kortrijk, Belgium).
Data format	Analysed
Experimental factors	Turbidity of the overnight broth cultures of *Salmonella* was adjusted to 0.5 McFarland Standard
Experimental features	Clustering of *Salmonella* strains using Pulsed-field gel electrophoresis (PFGE) fingerprints. The diameter of inhibition zones on agar was measured and interpreted as resistant by referring to breakpoints suggested by CLSI. The presence of antibiotic resistance and integrase genes were detected by PCR amplification.
Data source location	Perlis and Penang, the northern states of Malaysia
Data accessibility	Data are presented as Table 1 in this article, and Microsoft Excel Worksheet, which are provided as Supplementary data.

**Value of the data**

• The data on the presence of multidrug-resistant *Salmonella* in poultry and processing environment is a good indicator to extensive use of antibiotic in poultry.
• DNA fingerprinting will help in understanding the *Salmonella* contamination patterns.
• The data is a good indicator for the government to create a national surveillance program focusing on monitoring the antibiotic resistance profiles and DNA fingerprinting of foodborne *Salmonella* in poultry and processing environment.
• The data will aid in the discussion of the potential dissemination of antibiotic resistance genes in poultry and processing environment.

## 1 Data

Table 1 lists the antibiograms of multidrug-resistant (MDR) *S.* Corvallis, *S.* Brancaster and *S.* Albany strains isolated from poultry and processing environment in northern Malaysia. The Microsoft Excel Worksheet that is provided as Supplementary data (Table S1) for this article lists the antibiotic resistance and integrase genes harboured by these *Salmonella* strains, and the PFGE clustering of these strains.

**Table 1:** Antibiograms of MDR *S.* Corvallis, *S.* Brancaster and *S.* Albany strains isolated from poultry and processing environment in northern Malaysia.

Serovar	Antibiogram	aMDR	No. of isolates
*S.* Corvallis	S3-TE	−	1
	S-S3-TE	+	5
	AMP-S3-TE	+	1
	S-AMP-S3-TE	+	7
	S-KF-S3-TE	+	1
	S-CIP-S3-TE	+	1
	S-C-S3-TE	+	1
	S-SAM-S3-TE	+	1
	S-AMP-SAM-S3-TE	+	1
	S-AMP-C-S3-TE	+	3
	AMP-C-SXT-W-S3-TE	+	1
	S-AMP-NA-C-SXT-W-S3	+	1
	S-AMP-C-SXT-W-S3-TE	+	1
	AMP-NA-C-SXT-W-S3-TE	+	1
			
*S.* Brancaster	AMP-C-TE	+	1
	S-AMP-S3	+	2
	S-AMP-S3-TE	+	2
	S-AMP-C-TE	+	1
	AMP-C-S3-TE	+	3
	AMP-C-SXT-W-TE	+	2
	S-AMP-C-SXT-W-S3	+	1
	S-AMP-SXT-W-S3-TE	+	1
	AMP-C-SXT-W-S3-TE	+	10
	CN-AMP-C-SXT-W-S3-TE	+	1
	S-AMP-C-SXT-W-S3-TE	+	7
	S-AMP-NA-C-SXT-W-S3	+	1
	AMP-SAM-C-SXT-W-S3-TE	+	1
	S-AMP-SAM-C-SXT-W-S3-TE	+	2
			
*S.* Albany	S-S3-TE	+	1
	AMP-S3-TE	+	1
	NA-C-S3	+	1
	S-AMP-S3-TE	+	1
	AMP-NA-C-SXT-W-S3	+	6
	AMP-SAM-NA-C-S3-TE	+	1
	S-AMP-NA-C-SXT-W-S3	+	4
	S-AMP-C-SXT-W-S3-TE	+	1
	AMP-NA-C-SXT-W-S3-TE	+	11
	AMP-SAM-NA-C-SXT-W-S3-TE	+	4
	S-AMP-NA-C-SXT-W-S3-TE	+	3
	S-AMP-SAM-NA-C-SXT-W-S3	+	1
	S-AMP-SAM-NA-C-W-S3-TE	+	1
	S-AMP-KF-NA-SXT-W-S3-TE	+	2
	S-AMP-KF-NA-C-SXT-W-S3-TE	+	7
	S-AMP-SAM-NA-C-SXT-W-S3-TE	+	1
	CN-AMP-CIP-NA-C-SXT-W-S3-TE	+	1
	CN-S-AMP-NA-C-SXT-W-S3-TE	+	2
	CN-S-AMP-KF-NA-C-SXT-W-S3-TE	+	1
	S-AMP-KF-SAM-NA-C-SXT-W-S3-TE	+	1
	CN-S-AMP-CIP-NA-C-SXT-W-S3-TE	+	2

Abbreviations: CN, gentamicin 10 µg; S, streptomycin 10 µg; AMP, ampicillin 10 µg; CRO, ceftriaxone 30 µg; KF, cephalothin 30 µg; SAM, ampicillin-sulbactam 10/10 µg; CIP, ciprofloxacin 5 µg; NA, nalidixic acid 30 µg; C, chloramphenicol 30 µg; SXT, sulphamethoxazole/trimethoprim 1.25/23.75 µg; W, trimethoprim 5 µg; S3, sulphonamide 300 µg; TE, tetracycline 30 µg.

a Multidrug resistance (MDR) is defined as a resistance to more than two types of antibiotic [1]. +, MDR; −, non-MDR.

## 2 Experimental design, materials and methods

### 2.1 Salmonella strains

*Salmonella enterica* subsp. *enterica* strains used in this study were previously isolated from a total of 182 poultry and environmental samples collected from wet markets and small-scale processing plant located in Penang and Perlis, the northern states of Malaysia. Seventeen different *Salmonella* serotypes were isolated and the predominant serotypes were *S.* ser. Albany, *S.* ser. Brancaster and *S.* ser. Corvallis [2]. In this study, 114 representative strains of these three predominant serotypes, *S.* Albany (*n* = 53), *S.* Brancaster (*n* = 35) and *S.* Corvallis (*n* = 26), were randomly selected for the following studies described below.

### 2.2 Pulsed-field gel electrophoresis

PFGE was conducted according to the standard operating protocol described by PulseNet, CDC [3]. DNA of Salmonella strains were digested with 50 U of restriction enzyme *Xba*I (Vivantis, Malaysia), at 37 °C for 2 h. DNA electrophoresis was performed on 1% (w/v) agarose gel in a CHEF Mapper system (BioRad, Hercules, CA) with 0.5 × Tris-Borate EDTA buffer. The gel was run for 20 h at 14 °C using a linear ramp of 2.16–63.8 s at 6 V/cm. *Xba*I-digested *Salmonella* Braenderup H9812 was used as the DNA size marker. PFGE data were processed using Bionumerics software version 7.0 (Applied Maths, Kortrijk, Belgium) and clustering of the fingerprints was performed as previously described [4].

### 2.3 Agar disk diffusion assay

Agar disk diffusion assay was performed by using commercially available antibiotic disks (Oxoid, Basingstoke, UK) on Mueller-Hinton agar (Oxoid, Basingstoke, UK) according to the guidelines of the CLSI [5]. *Escherichia coli* ATCC 25922 was employed as a positive control. The diameter of inhibition zones was measured and interpreted as resistant by referring to breakpoints suggested by CLSI [5]. *Salmonella* strains classified as intermediate susceptible on the basis of inhibition zone were considered as sensitive for resistance spectrum. Strain that was resistant to more than two types of antibiotic was regarded as MDR [1].

### 2.4 Detection of antibiotic resistance and integrase genes using PCR

Genomic DNA was prepared by using phenol-chloroform extraction method, adapted from Santos et al. [6] with modifications. PCR reaction mixtures were prepared as described by Benacer et al. [7] and amplification was performed using a TProfessional Standard Gradient96 Thermocyler (Biometra, Germany). The presence of integrase genes and antibiotic resistance genes conferring to resistance against β-lactams, aminoglycosides, tetracyclines, quinolones, sulphonamides and chloramphenicol, was detected using primers previously reported [8].
